# Plasticity in nest site choice behavior in response to hydric conditions in a reptile

**DOI:** 10.1038/s41598-020-73080-6

**Published:** 2020-09-29

**Authors:** J. Sean Doody, Jessica McGlashan, Harry Fryer, Lizzy Coleman, Hugh James, Kari Soennichsen, David Rhind, Simon Clulow

**Affiliations:** 1 Department of Integrative Biology, University of South Florida, St. Petersburg, FL 33701 USA; 2 School of Environmental and Life Sciences, University of Newcastle, Callaghan, NSW 2308 Australia; 3 School of Health Sciences, University of Western Sydney, Locked Bag 1797, Penrith, NSW 2751 Australia; 4 Department of Biological Sciences, Macquarie University, Balaclava Rd., Macquarie Park, NSW 2109 Australia; 5 Department of Anatomy and Developmental Biology, Monash University, Clayton, VIC 3800 Australia

**Keywords:** Ecology, Evolution, Zoology

## Abstract

Natural selection is expected to select for and maintain maternal behaviors associated with choosing a nest site that promotes successful hatching of offspring, especially in animals that do not exhibit parental care such as reptiles. In contrast to temperature effects, we know little about how soil moisture contributes to successful hatching and particularly how it shapes nest site choice behavior in nature. The recent revelation of exceptionally deep nesting in lizards under extreme dry conditions underscored the potential for the hydric environment in shaping the evolution of nest site choice. But if deep nesting is an adaptation to dry conditions, is there a plastic component such that mothers would excavate deeper nests in drier years? We tested this hypothesis by excavating communal warrens of a large, deep-nesting monitor lizard (*Varanus panoptes*), taking advantage of four wet seasons with contrasting rainfall amounts. We found 75 nests during two excavations, including 45 nests after a 4-year period with larger wet season rainfall and 30 nests after a 4-year period with smaller wet season rainfall. Mothers nested significantly deeper in years associated with drier nesting seasons, a finding best explained as a plastic response to soil moisture, because differences in both the mean and variance in soil temperatures between 1 and 4 m deep are negligible. Our data are novel for reptiles in demonstrating plasticity in maternal behavior in response to hydric conditions during the time of nesting. The absence of evidence for other ground-nesting reptile mothers adjusting nest depth in response to a hydric-depth gradient is likely due to the tradeoff between moisture and temperature with changing depth; most ground-nesting reptile eggs are deposited at depths of ~ 2–25 cm—nesting deeper within or outside of that range of depths to achieve higher soil moisture would also generally create cooler conditions for embryos that need adequate heat for successful development. In contrast, extreme deep nesting in *V. panoptes* allowed us to disentangle temperature and moisture. Broadly, our data suggest that ground-nesting reptiles can assess soil moisture and respond by adjusting the depth of the nest, but may not, due to the cooling effect of nesting deeper. Our results, within the context of previous work, provide a more complete picture of how mothers can promote hatching success through adjustments in nest site choice behavior.

## Introduction

Most traits are subject to natural selection, and in theory populations have multiple evolutionary options for solving problems. For example, a rodent mother can choose a birthing site that is optimal for rearing young, but if she makes a poor choice she can compensate by moving her young to a more suitable site. Both of these maternal behaviors are subject to selection and can thus theoretically evolve to promote offspring survival^1^. Most animals are oviparous ectotherms that do not provide parental care, however, and in these animals the mother abandons the immobile eggs^2^. She therefore must conceal them from predators and predict the environmental conditions subsequently experienced by developing embryos. Offspring survival in these animals is thus more reliant on one set of behavioral traits—oviposition site choice or nest site choice. We would expect natural selection to produce and maintain such behaviors to promote successful hatching, provided that maternal survival is not compromised during nesting^3,4^.

Maternal behaviors can thus act as ‘filters’ against selective forces in nature such as predation, freezing, overheating, and desiccation. Indeed there are numerous studies revealing that hatching success can be influenced by oviposition site choice in birds, amphibians, reptiles and insects^1,3,5^, and there is evidence that such behaviors are heritable^6^ (but see^7^). A stereotypical example is a reptile that excavates and backfills a nest in the ground and then abandons the nest, leaving the eggs to the vagaries of prevailing weather conditions and olfactory-driven predators for weeks to months.

Developing reptile embryos in situ require suitable ranges of temperature and moisture, along with sufficient gas exchange, to complete embryogenesis and hatch successfully^8^. Our knowledge of the effects of these factors on developing embryos is dominated by temperature effects; the mean and variance in temperatures across various spatial scales can affect developmental rate, incubation period, hatching success, hatchling phenotypes and growth rates, and thus, very likely, offspring fitness^9–11^. In turn, nest site choice can have strong direct effects on nest temperatures in nature (e.g.,^12^), thus providing a link between nest site choice and fitness-related traits of offspring^13^. Moreover, clinal variation in nest site attributes across environmental (temperature) gradients in some reptiles is suggestive of temperature-driven, among-generation adaptation in nesting behavior^14,15^. For example, lizards in cooler climates nested under more open canopies, at shallower depths, and at warmer aspects than those in warmer climates^14,16,17^.

In contrast to temperature effects, we know little about how soil moisture contributes to successful hatching of reptiles in nature, and thus how the hydric environment shapes the evolution of nest site choice behaviors, despite laboratory and field experiments revealing limits in some species^18–21^. There is ample evidence that extreme hydric conditions can reduce hatching success in the laboratory^22–25^, and a few field studies have demonstrated links between nest site moisture and hatching success and fitness-related traits under natural conditions^26,27^. Flooded or saturated eggs can result in embryonic mortality^22,28–33^, and embryos can desiccate and perish during very dry soil conditions within or among nests and within or among years^30,34–37^. Despite the link between hydric conditions and hatching success, few studies have quantitatively coupled this link with nest site choice variables in nature (but see^26,27,30,38^).

Although among-generation adaptation may be the primary way in which nesting mothers offset environmental gradients, maternal behavior can also respond to changes in environmental conditions through phenotypic plasticity in nest site choice. For example, if a ground-nesting reptile mother is sampling substrate temperatures when making the decision of where to nest, variation in environmental conditions (i.e., due to a particularly hot year, season, or day) could cause her to nest deeper, in a more shaded site, or at a cooler aspect.

The recent revelation of exceptionally deep nesting in two large monitor lizards in the wet-dry tropics and deserts of Australia underscored the potential for the hydric environment in shaping the evolution of nest site choice. The yellow-spotted monitor (*V. panoptes*) and Gould’s monitor (*V. gouldii*) excavate deeper nests than any other ground-nesting vertebrate, depositing their eggs up to 4 m below the surface in communal nesting warrens^39–42^. Deep nesting in these species appears to be a response to hydric conditions: embryos require very long (~ 8 months) developmental periods that must span the long, harsh dry season, and nesting at shallower depths would likely result in the desiccation of embryos^39,41,42^. In support, mean and variance in soil temperatures in warrens were very similar among depths in the 1–4 m range^39^, and nest depth was inversely proportionate to annual wet season rainfall across a rainfall gradient^42^.

Although the deep-nesting monitor lizards provided a novel study system for understanding the importance of the hydric environment on the evolution of nest site choice, a critical missing piece of the puzzle is the role of phenotypic plasticity in nest site choice. If deep nesting is an adaptation to (extreme) dry conditions, is there a plastic component such that mothers would excavate deeper nests in drier years but conserve energy and nest shallower in wetter years? We took advantage of two 4-year periods with contrasting rainfall amounts during the nesting season to compare nest depths of *V. panoptes* nesting warrens. We predicted that mothers would nest deeper in drier years to reduce the probability of egg desiccation (and ensure successful hatching). We also compared clutch sizes between the two periods, predicting lower clutch sizes following the drier nesting seasons due to the possibility of reduced energy acquisition in mothers. We discuss our findings within the context of the evolution of nest site choice and phenotypic plasticity.

## Materials and methods

### Study area, species and study period

The study area was Gogo Station, near Fitzroy Crossing in the southwest Kimberley region in Western Australia (− 18.212761°S, 125.861943°E, elev. 127 m). The area is situated in the Wet-dry tropics, with a distinct dry season during which time very little rain falls from May to October^43^. The landscape is in open savannah woodland, and the station is managed for cattle and is flanked by the Margaret River. The banks and floodplain of that river is inhabited by the yellow-spotted monitor, *Varanus panoptes*, a large carnivorous lizard that occupies a top predator position in the food web in riparian areas, floodplains, and dry riverbeds in tropical and western Australia^44–46^. Like other large monitor lizards, it has a notably long incubation period, ranging from 210 to 365 days^47^. Although information on the timing of oviposition in this species is scarce, *V. panoptes* lays eggs in the late wet season/early dry season, with hatching occurring in the following wet season^47,48^. It excavates nesting burrows communally in warrens, depositing its eggs deeper (up to 4 m) than any other vertebrate, except perhaps its closest relative, *V. gouldii*^39,41,42^.

We excavated the same nesting warren in June in both 2013 and 2019, and an additional six warrens excavated in 2013 as part of an earlier study^39^. The warrens, which were generally around 10–100 m^2^, were along the Margaret River and generally received an abundance of sunlight but were partially shaded by a few trees and/or large shrubs. The surrounding areas were grassy but intense grazing, especially evident in 2019, reduced the grass cover considerably. The warrens themselves were generally free of vegetation, presumably due to the burrowing activity of the lizards, but perhaps also influenced by inquilines. The soils were typically sandy loam.

### Warren excavation and location of nests

**Figure 1:**
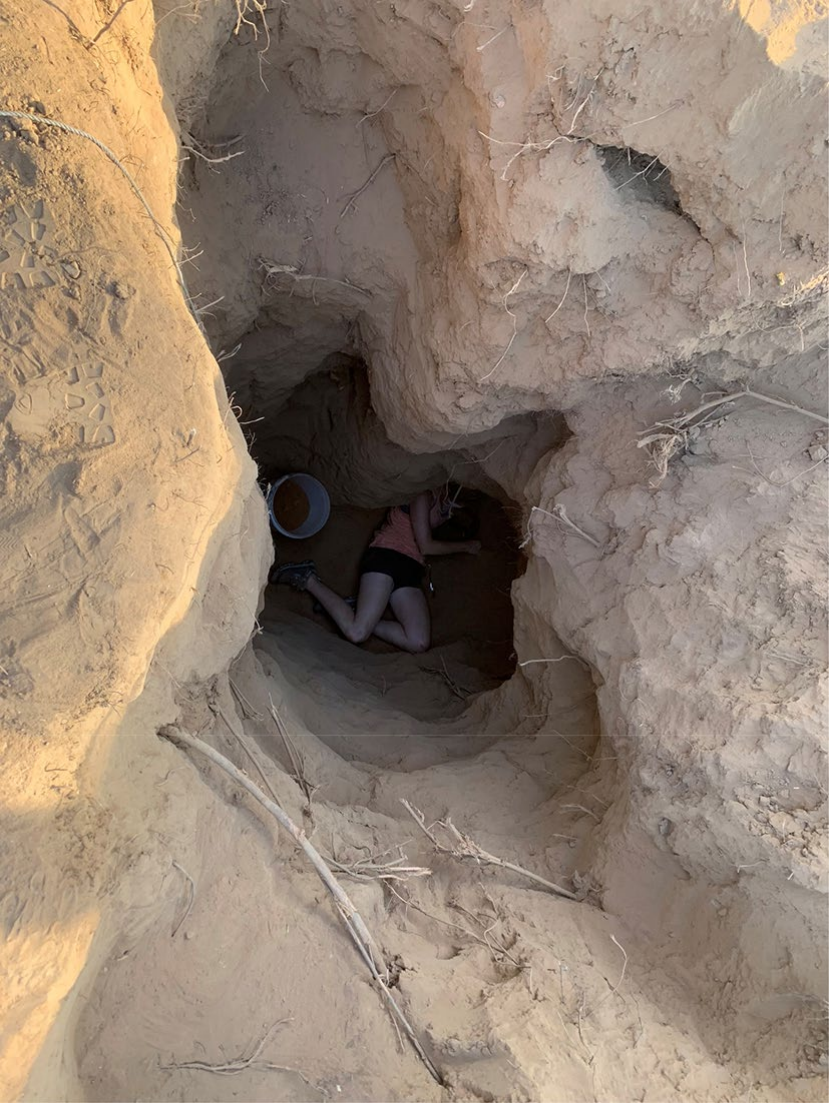
Field assistant in an excavated portion of a *V. panoptes* nesting warren. Note burrow ca. 30 cm deep in the top right hand part of photograph. Photo by S. Doody.

Warrens were excavated by hand with shovels, pics, trowels and spoons to prevent damage to eggs and to facilitate carefully following individual burrows to their termini (Fig. 1). A team of 4–6 persons was generally required for 5–7 days to excavate a warren. Excavations proceeded by following individual burrow entrances until the burrow either terminated or led to a nest. Once the excavation became > 2 m deep we used a bucket on a rope to remove soil (Fig. 1). Although the warrens are communal, each nests is deposited in the terminus of a unique burrow. For a few nests we had depths but not clutch sizes due to a nest containing only eggshell fragments or another burrow intercepting the nest causing eggshells to be pushed out of the nest by conspecific mothers or other animals. We did not know if nests across years were from the same mothers. When a nest was discovered we carefully measured straight line depth from the bottom of the nest to the surface with a tape measure. The presence of eggs reflected nesting during the wet season or early dry season that year, while the presence of hatched eggshells indicated nesting in the wet season/early dry season during the previous ~3 year, based on the number of nests found. Eggs and eggshells were counted to determine clutch size. Eggs were returned to their original depths prior to back-filling the warren. Eggs were handled with care as they are prone to easily break, and we were careful to not rotate eggs during the time they were out of the ground.

### Treatment of the data and statistical analyses

Nest depth was analyzed against monthly rainfall for February and March = the 2 months in which nesting has been recorded to occur at the site. Nests with eggs found in 2013 were thus considered to have been deposited during the past February–March (2013) while nests with hatched eggshells found in 2013 were considered to reflect nests deposited during the previous three wet seasons (2010–2012). Similarly, nests with eggs found in 2019 were considered to have been deposited during February–March (2019) while nests with hatched eggshells found in 2019 were considered to reflect nests deposited during the three previous wet seasons (2016–2018). Rainfall values used for 2013 and 2019 nests with eggs were thus the mean monthly rainfall amounts for February–March of each respective year; for nests with eggshells we used the mean monthly rainfall amount for the three previous wet seasons, as we could not determine, from eggshells, which year the nests were deposited. Rainfall data were obtained from the Australian Bureau of Meteorology (www.bom.gov; accessed 1 February 2020).

Each data set was depicted graphically against annual wet season rainfall as a continuous variable to visually inspect the fit of the data and shape of the curve and was modelled according to the distribution and fit of the data. The modelling was carried in SAS (version 9.4) using the glimmix procedure, with fixed effects for annual wet season rainfall and a random effect for nesting warren to account for variability between warrens that was not explained by wet season rainfall.

Nest depth was found to be normally distributed so a Linear Mixed Model (LMM) was used to model the effect of wet season rainfall on nest depth.

Clutch size was a count variable and thus a Generalised Linear Mixed Model (GLMM) was used to examine the effect of wet season rainfall on clutch size using the Poisson distribution with identify link function. A visual inspection of the data (Fig. 3) showed a positive relationship between clutch size and increasing annual wet season rainfall, although with visual evidence of either a linear relationship or a possible threshold effect that might be reflecting a sigmoid relationship. As such, two GLMM models were built for clutch size: one with annual wet season rainfall as a continuous variable (indicative of a linear effect) and the other with annual wet season rainfall as a categorical variable (testing the possibility that a threshold effect might be a better description than the linear effect). Akaike Information Criteria (AIC) were used to compare the two models, with an AIC difference of > 2 between the models suggesting a better fit of the data of one model over the other (with the smaller of the two AICs being the better fit^49^). In the case of the categorical model being a better fit, differences in the estimated marginal means were used to further explore the data to assess where along the curve significant changes occurred (i.e. where the threshold effect took place). All reported means for clutch size are fitted model means.

## Results

### Descriptive and climate data

We found 75 nests during the two excavations, including 45 nests in 2013 and 30 nests in 2019. In 2013, nine of the nests contained eggs deposited during the current reproductive season and 36 contained eggshells from nests deposited in previous years, while in 2019, seven of the nests contained eggs deposited during the current reproductive season and 23 contained eggshells from nests deposited in previous years. The nests in 2013 were from seven warrens compared to one warren in 2019 (the warren in 2019 was one of those excavated in 2013).

**Figure 2:**
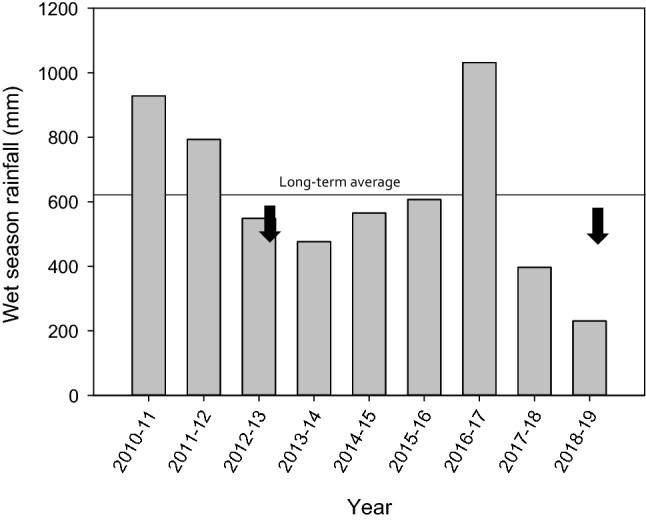
Rainfall during the wet seasons (November–April) prior to nesting during two excavations, one in 2019 (after two very small wet seasons) and one in 2013 (after two average to large wet seasons). Data are from Fitzroy Crossing Aeroport (Bureau of Meteorology), 29 km west of the study site. Arrows denote the timing of the excavations. Long-term average is from 24 years of data (1997–2020).

The magnitude of February–March rainfall varied markedly over the years relevant to the present study (Fig. 2). For example, for nests deposited in 2010–2013 mean rainfall amounts were 240 mm (eggs, 2013) and 294.7 mm (eggshells, 2010–2012 combined), while for those deposited in 2016–2019 rainfall amounts were 101 mm (eggs, 2019) and 155 mm (eggshells, 2016–2018 combined).

### Influence of wet season on nest depth

**Figure 3:**
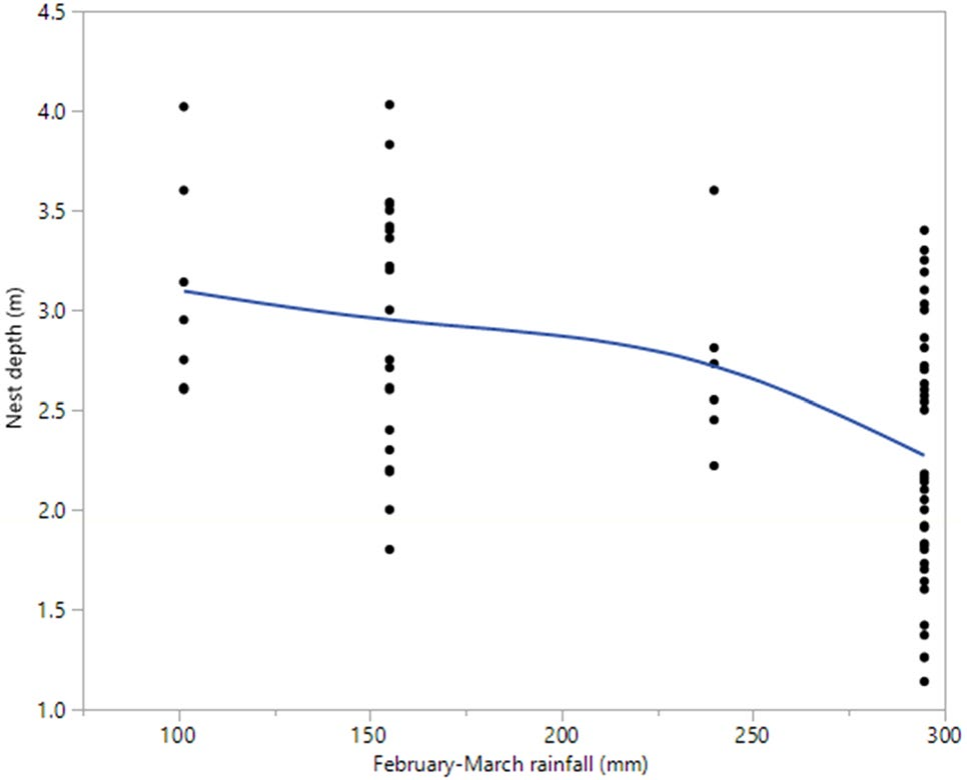
Nest depth plotted against February–March rainfall. Data are from eggs and eggshells found in 2019 (after periods of less rainfall) and those found in 2013 (after periods of more rainfall). See “Materials and methods” section for details on treatment of the data.

Nest depth was significantly inversely related to the magnitude of wet season rainfall (F_1,67_ = 5.39; *P* = 0.02; Fig. 3). The mean nest depth decreased from 3.1 ± 0.54 m (SD) in the smallest wet season (2019; 101.4 mm) to 2.27 ± 0.59 m in the largest wet season (average of 2010–2012; 294.7 mm).

### Influence of wet season on clutch size

**Figure 4:**
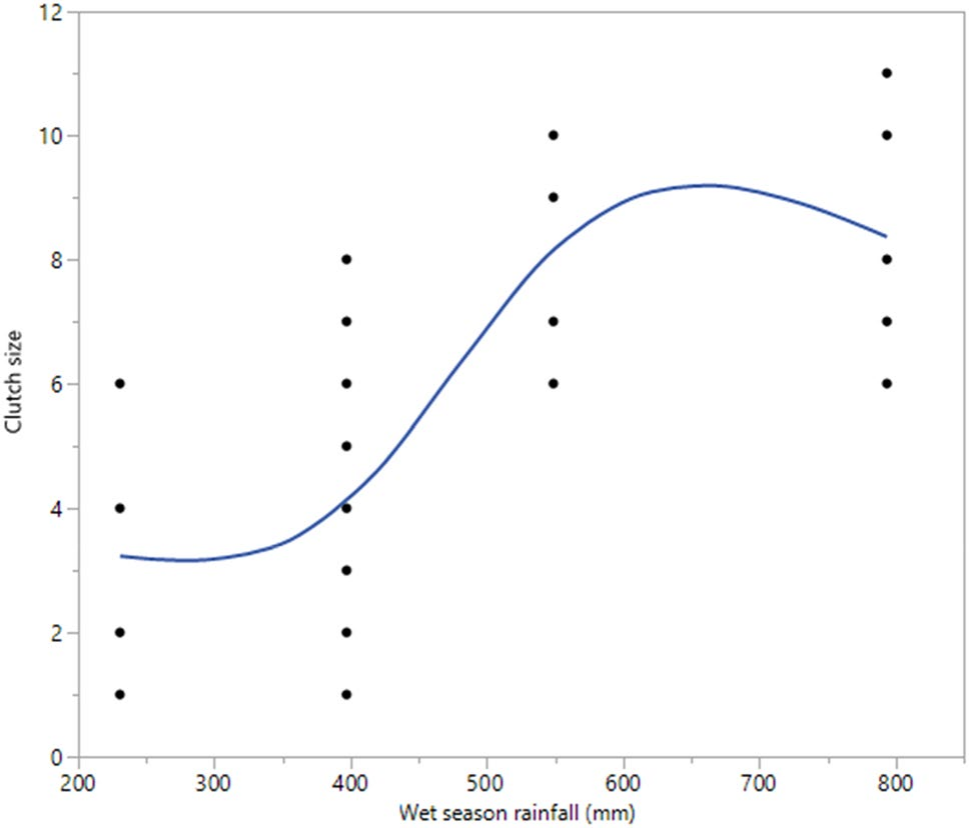
Clutch size plotted against mean monthly rainfall (November–April) for the previous wet season. Data are from eggs and eggshells found in 2019 (after periods of less rainfall) and those found in 2013 (after periods of less rainfall). See “Materials and methods” section for details on treatment of the data.

Clutch size was significantly positively related to the magnitude of wet season rainfall, with both the continuous (F_1,49_ = 42.31; *P* < 0.001) and categorical (F_3,47_ = 15.28; *P* < 0.001) models showing highly significant effects (Fig. 4). The model AICs (continuous = 242.65, categorical = 240.41; difference = 2.24) suggest that the categorical model was a better fit, providing support for a threshold effect along a sigmoidal curve rather than a linear effect. Further support for the threshold effect was provided by the differences in the estimated marginal means. Mean clutch sizes did not differ significantly between the two smallest wet seasons (mean clutch size = 3.3 ± 1.70 (SD) and 4.1 ± 0.92 eggs for annual rainfall of 230 mm and 397 mm respectively; t = 0.97, *P* = 0.35) or two largest wet seasons (mean clutch size = 8.3 ± 1.54 (SD) and 8.4 ± 1.33 eggs for annual rainfall of 549 mm and 794 mm respectively; t = 0.01, *P* = 0.99), but there was a large and significant increase in clutch size between the two smallest to the two largest wet seasons (e.g. from 397 to 549 mm, mean clutch size doubled from 4.1 ± 0.85 (SD) to 8.3 ± 1.69 eggs per clutch; t = 5.55, *P* < 0.01). This suggests a significant threshold effect on clutch size for annual wet season rainfall somewhere between ca. 397 mm and 549 mm (Fig. 4).

## Discussion

We present indirect but compelling evidence for phenotypic plasticity in nest depth chosen by mothers across years with marked differences in wet season rainfall, a proxy for soil moisture. Mothers chose to nest deeper in drier years. Our data are novel for reptiles in demonstrating plasticity in maternal behavior as a result of hydric conditions during the time of nesting. The lack of similar findings prior to our study are likely due to the trade-off between moisture and temperature with depth in most reptiles; extreme deep nesting in *V. panoptes* allowed us to disentangle temperature and moisture. Our data suggest that ground-nesting reptiles can assess soil moisture and respond by adjusting the depth of the nest, but may not, due to the cooling effect of nesting deeper. This finding, in tandem with previous findings of apparent among-generation adjustments in nest depth across a rainfall gradient, provide a more complete picture of how mothers can promote hatching success through adjustments in nest site choice behavior. Finally, we also revealed marked variation in clutch size associated with the magnitude of wet season rainfall, likely caused by variation in energy acquisition or size-mediated mortality of mothers caused by invasive cane toads (see below).

Although the evolution of the amniotic egg released oviparous reptiles and their kin from laying eggs in water or wet places, reptile embryos typically require moist conditions for successful development and hatching. Mothers in the present study responded to drier conditions by nesting up to 73% deeper (3.1 m in the largest wet season compared to 2.27 m in the smallest wet season) (Figs. 2, 3). The vast majority of the nests were from the same warren, eliminating possible variation in local microclimate or soil type that might influence nesting mothers (moreover, warren was added as a random effect in the models). The variation in nest depths was not likely a result of variation in temperature across years because both the mean and variance in nest temperature remain virtually unchanged as you descend from 1 to 4 m deep in *V. panoptes* nests (see Fig. 4 in^39^). We can think of no other reason why nest depth would vary significantly and consistently with wet season rainfall, and our interpretation is supported by the species nesting deeper in more arid locations across a rainfall gradient^39,41,42^ (and T. Madsen, unpubl. data). Although we did not measure soil moisture, *V. panoptes* nest in the late wet season/early dry season and eggs incubate for 8 months throughout the long dry season, during which time an obvious soil moisture gradient with depth exists^39^.

**Figure 5:**
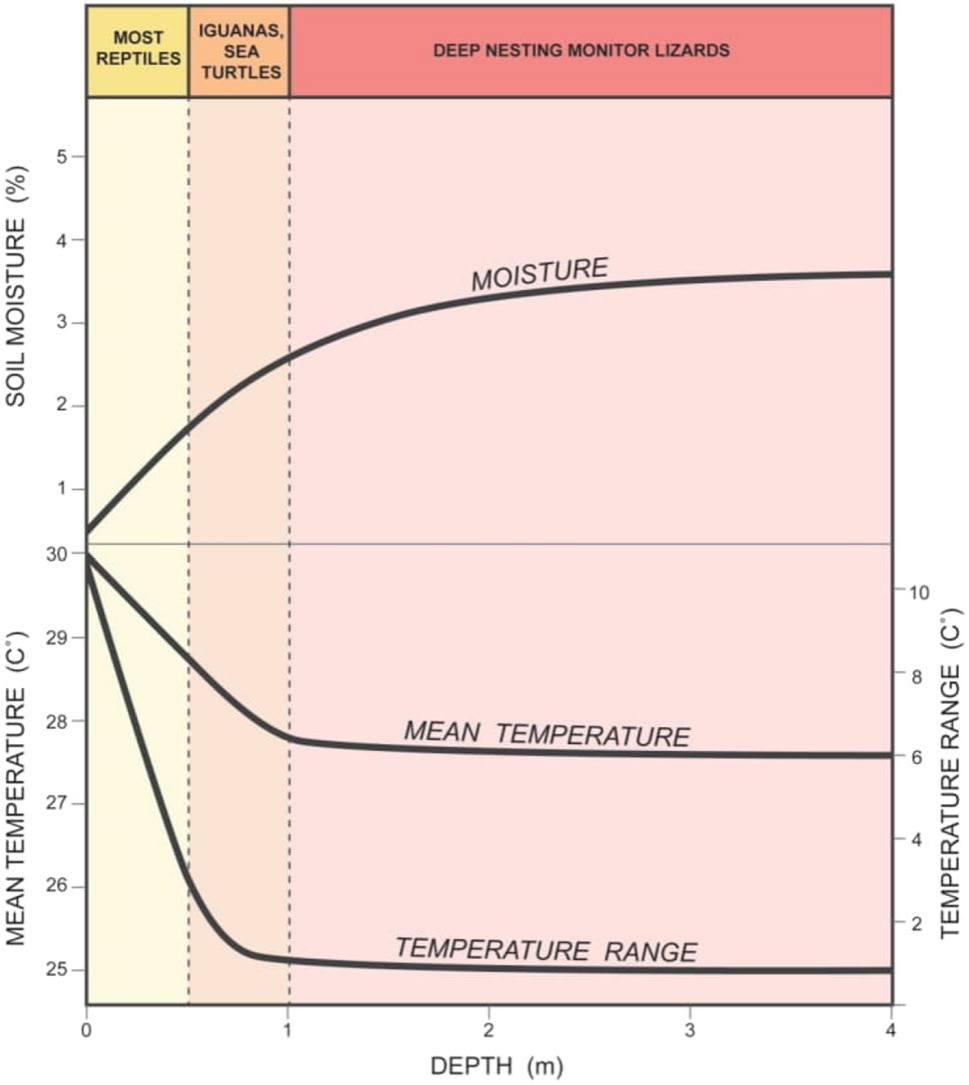
Relationships between soil moisture (top) and soil temperatures (bottom) with depth for ground-nesting reptiles, based on data taken from *V. panoptes* nesting warrens^39,46^. Most ground-nesting reptiles nest between 2–30 cm deep, where there is often a strong inverse relationship between soil moisture and both mean and daily range in soil temperatures. A few larger species (e.g., iguanas, sea turtles) deposit nests at depths of up to 1 m, but only deep-nesting monitor lizards nest 1–4 m deep, and in these nests moisture and temperature (both mean and daily range) become uncoupled because the change in temperatures with depth through 1–4 m is negligible. There are two caveats here: (1) a rainfall event can temporarily reverse the soil moisture-depth gradient shown; and (2) the mean temperature-depth gradient shown is restricted to seasons in which mean air temperature is generally higher than mean soil temperature when considering depths > 1 m (i.e., during spring and summer).

Studies demonstrating the hydric environment as the principle cause of variation in nest depth are absent. Water dragons (*Intellagama lesueurii*) nested in shallower depths at higher elevations across a 700 m gradient^16^; reduced hatching success at the highest elevation may have reflected desiccation, but exposure to lethally high temperature could not be ruled out. Nest depth decreased over a 10-year period in the lizard *Acritoscincus (Bassiana) duperreyi*, but the authors implicated increased air temperatures as the cause and did not examine soil moisture or rainfall^50^. Similarly, after finding an inverse relationship between vegetative cover and nest depth in the iguana *Cyclura cychlura*, Iverson et al.^30^ suggested such plasticity may have reflected the lizards’ choice of depths with preferred temperatures. However, because shallower nests are often both hotter and drier (Fig. 5), plasticity in depth could have been due to temperature, moisture or both. Our knowledge of nest site choice in reptiles is overwhelmingly based on studies of turtles, however, because they are often large, conspicuous animals that nest in open areas and leave tracks in the sand along watercourses. As with lizards, turtle researchers have found that nest depth influences hatching success across years or habitat types (e.g.,^34,51^). Painted turtle (*Chrysemys picta*) mothers at a warmer latitude nested deeper than those at a cooler latitude^52^, possibly to compensate for higher ambient temperatures at the southern edge of the species range^53,54^, and the shallow-nesting and small turtle *Kinosternon baurii* nested closer to grass tussocks and other herbaceous vegetation than random sites in subtropical Florida, USA; experimental eggs experienced higher survival in the former, probably due to eggs overheating, although desiccation could not be ruled out^51^.

Why hasn’t the response of digging deeper nests under drier conditions been revealed for reptiles previously? The most likely answer is that temperature confounds the relationship between depth and moisture such that reptile mothers may typically not be able to nest deeper under drier conditions without a concomitant reduction in nest temperatures. Most ground-nesting reptile eggs are deposited at depths of ~ 2–25 cm^40^; moving eggs within that range would have considerable thermal consequences, especially in daily fluctuations that explain much of the heating in shallow reptile nests (Fig. 5; ^55^). In other words, nesting incrementally deeper to achieve higher soil moisture would also create cooler conditions for embryos that need adequate heat for successful development. The opposite is also true: nesting more superficially to achieve higher nest temperatures, which appears to have been the case with the lizard *Intellagama lesueurii* at higher elevations^16^, can result in drier nest conditions. In that study eggs from the highest elevation nests appear to have desiccated, although high-temperature mortality could not be ruled out. Another option to avoid dry conditions is to switch nesting habitat: the skink *Mabuya (Trachylepis) maculilabrus*, which nests in the ground during the rainy season, apparently nests in palm shoots during the dry season^56^.

In contrast, extreme deep nesting in *V. panoptes* allowed us to disentangle temperature and moisture (Fig. 5). In this species nests are so deep as to virtually eliminate daily fluctuations (mean nest depth varied from 1 to 4 m in the present study), and the mean nest temperature is essentially the same between 1 and 4 m deep (Fig. 5; ^39^). Thus, *V. panoptes* could adjust nest depth to offset changes in hydric conditions without compromising nest temperatures (Fig. 5). There are two caveats here: (1) a rainfall event will temporarily reverse the soil moisture-depth gradient shown; and (2) the mean temperature-depth gradient shown is restricted to seasons in which mean air temperature is generally higher than mean soil temperature (spring, summer). Indeed, most of the world’s reptiles nest in spring or summer^57,58^ (but see^59^ for some exceptions).

Another reason for the paucity of plasticity in depth data under different hydric regimes is the formidable challenge of measuring soil water potentials in nests in situ (see examples in^21,60^). In some cases we can use a proxy for soil water potential or soil moisture, however, such as variation in rainfall among locations or among years. Our study found an apparent proxy for soil moisture—wet season rainfall—in a climate with an extreme dry period. Very little rain falls between May and October in much of the Australian wet-dry tropics (e.g.,^61^). Our study thus suggests that ground-nesting reptiles can assess soil moisture and respond by adjusting the depth of the nest, but may not, due to the cooling effect of nesting deeper. A third reason for limited nest depth variation is that most studies on reptile nesting are in moist climates; future attempts to reveal plasticity in nest site choice in response to hydric conditions should focus on populations that inhabit dry ecosystems or those with an extreme dry season. Finally, the difficulty in finding lizard and other reptile nests in nature no doubt contributes to the paucity of plasticity in depth data induced by hydric conditions.

The marked reduction in clutch sizes from nests found in 2019 compared to those found in 2013 (Fig. 4) may be due to lower energy acquisition related to reduced food availability during dry years. Mean clutch sizes found in 2019 were 3.3 or 4.0 when considering eggs and eggshells, respectively. These values are 48–60% lower than those for 2013 (means = 7.7–8.3 eggs). The wet season during nesting in 2018–2019 was one of the lowest on record (230.6 mm) and the year prior that was also very low (397 mm) compared to the long-term 23-year average of 621.5 mm (Fig. 2). In contrast, wet season rainfall was near average for 2012–2013 (548.8 mm) and above average the previous year (793.6 mm). The 2-year total rainfall for 2011–2012/2012–2013 was more than double that of 2017–2018/2018–2019 (1343 mm vs. 624 mm). Although some of the eggshells found in 2019 could be from nests deposited during a large wet season in 2016–2017 (963.9 mm), this number would be a relatively small portion of the total nests found that year. Thus, it appears that very small wet seasons may have resulted in lower food uptake for *V. panoptes* mothers, and this was manifest in reduced clutch sizes. An increase in clutch size due to higher rainfall has been documented in various animals (e.g.,^62,63^), including experimentally in lizards^64^.

There is another possible reason for low clutch sizes in nests found in 2019. Invasive cane toads, which decimate *V. panoptes* via lethal toxic ingestion^45^, arrived at the site in 2018 (indeed, we found a toad in one of the burrow entrances in 2019). Because the first-arriving toads are large adults that may be too large for (some) *V. panoptes* mothers to feed on, it is possible that mainly larger females had begun to succumb to cane toads by 2018–2019, leaving a nesting sample that was biased towards smaller mothers. If this was the case, then we might expect smaller clutch sizes, because clutch size is generally correlated with female body size in lizards^19^. Biologists have noticed that when toads first arrive in an area the larger individual monitor lizards disappear rapidly, resulting in a population of relatively smaller individuals in some localities where the species persists (Doody et al., in press, K. Christian, pers. comm.). This mortality-based shift in body size could also be due to the larger males feeding more aggressively than females. For example, compared to larger individuals, smaller monitors (*V. varius*) were more cautious in investigating novel baits and swallowed the baits more slowly^65^.

The shape of the best-fitting curve describing the relationship of clutch size to wet season rainfall suggests a possible ‘floor and ceiling’ for the effect of annual wet season rainfall on clutch size. There was little effect of wet season magnitude on clutch size at annual rainfall amounts < 400 mm and > 550 mm, suggesting that food availability is mainly affected by rainfall amounts between those values. However, this hypothesis needs confirmation with a greater number of nesting years and more direct measurements of food consumption (e.g., field energetics) or body condition. A generalist carnivore, *V. panoptes* feeds on invertebrates, vertebrates, and eggs^66^, and its aestivation during the mid-to-late dry season is likely related to a lack of food rather than temperatures during the tropical winter^67^.

In summary, despite much attention given to phenotypic plasticity in the responses of reptiles to temperature (see reviews in^68,69^), natural selection in response to hydric conditions is less understood. Yet precipitation, and thus hydric conditions, may be a better predictor of variation in natural selection across populations of terrestrial animals^70^. Our unique study system allowed us to disentangle temperature and moisture in the nests of a reptile, thereby exposing the apparent plasticity in nest site choice, via nest depth, in that system. Future studies should further examine the spatial and temporal variation in nest depth in this species and other deep-nesting reptiles to confirm the importance of nest moisture without the confounding factor of temperature.
